# A geo-additive spatial analysis of condom use among women aged 15–49 in Rwanda

**DOI:** 10.3389/fpubh.2025.1635694

**Published:** 2025-07-31

**Authors:** Mkhombiseni Zamani Sithole, Jesca Mercy Batidzirai, Ashenafi Argaw Yirga, Retius Chifurira

**Affiliations:** ^1^School of Agriculture and Science, University of KwaZulu-Natal, Pietermaritzburg Campus, College of Agriculture, Science and Engineering, Pietermaritzburg, South Africa; ^2^Center for Policy, Planning, and Evaluation, DC Department of Health, District of Columbia, United States; ^3^School of Agriculture and Science, University of KwaZulu-Natal, Westville Campus, College of Agriculture, Science and Engineering, Durban, South Africa

**Keywords:** condom use, geo-additive model, spatial analysis, women, Rwanda

## Abstract

**Background:**

Condom use in Rwanda is a significant public health topic, especially in efforts to prevent sexually transmitted infections (STIs), including HIV/AIDS, unplanned pregnancies and other health problems such as cervical cancer. In Rwanda, the government and various organizations actively promote condom use through comprehensive sexual and reproductive health initiatives such as sexual health education and condom distribution in schools, communities and public places. Despite these efforts, many individuals still fail to adopt consistent condom use, leading to significant health consequences. To enhance health and condom promotion programs, it is crucial to understand the factors contributing to low condom use and to identify regions with the lowest uptake.

**Methods:**

This study analyzes data from the 2019/2020 Rwanda Demographic and Health Survey (RDHS) to investigate the prevalence and determinants of condom use during the last sexual intercourse among reproductive-aged women in Rwanda. A geo-additive model was employed to account for geographical variation in condom use, enabling the identification of regional disparities and spatial patterns. These methodological approaches provide a robust framework for understanding individual and contextual factors influencing condom use in Rwanda.

**Results:**

The findings revealed a 10.8% prevalence of condom use and highlighted significant regional disparities in usage patterns. Women who reported living with a man had significantly lower odds of condom use compared to those not living with a man (AOR = 0.07, 95% CrI: 0.06–0.09). Those with primary education had significantly higher odds of condom use compared to those with no education (AOR = 1.39, 95% CrI: 1.04–1.84). Central and Northern districts, such as Ruhango and Musanze, showed positive structured spatial effects, suggesting regionally correlated factors promoting condom use. Unstructured spatial effects highlighted significant district-specific variations, with areas like Ruhango and Rusizi exhibiting higher odds of condom use, while Nyamagabe and Gasabo had lower odds.

**Conclusion:**

Spatial disparities in condom use suggest a need for region-specific interventions, particularly in the Southern and Northern regions of Rwanda. Efforts should focus on enhancing education access and economic empowerment while addressing cultural and relational barriers to condom use in cohabiting relationships.

## Background

Condom use is a critical public health concern, particularly in efforts to prevent sexually transmitted infections (STIs), including HIV/AIDS, unplanned pregnancies, and cervical cancer (1). In Rwanda, the government and various organizations actively try to promote condom use as part of comprehensive sexual and reproductive health strategies (2, 3). National campaigns advocate for condom distribution and education in schools, communities, and workplaces. Despite a declining HIV prevalence in Rwanda, new HIV and STD infections and ongoing challenges persist (4).

Based on evidence from longitudinal studies, the effectiveness of condoms in preventing HIV is estimated to be 80–85%, although it may be as high as 95% when used consistently and correctly (5). The United Nations 90–90-90 goal is to end the AIDS epidemic as a public health threat by 2030, incorporating efforts that include increasing access to prevention tools such as condoms, PrEP, and other measures (6). Condoms are one of the most easily available, flexible and cost-effective health commodities in Rwanda, which, in addition to STI/HIV prevention, may be used as a contraceptive (7). It is, therefore, integral to family planning programs, helping to manage population growth and improve maternal health outcomes. Despite all efforts by the government, some people still do not use condoms.

Women of reproductive age are on the receiving end where they are the ones who are at a higher risk of acquiring HIV, STI, cervical cancer and unwanted pregnancies. They are the ones who suffer higher levels of poor physical and mental health, poor psychological health, depression, anxiety, and phobias, and are more likely to harbor thoughts of suicide and attempted suicide (8). In addition, young women might suffer consequences such as school dropout and disruptions in their career paths (8). In this light, women’s behavior pertaining condom use needs to be established in order to identify ways to support them effectively. Further, condom use prevalence in Rwanda may vary with geographical locations, possible due to unmeasured variations in cultures, norms, access to condoms from various health facilities and sex education (9, 10). For effective interventions on sexual health promotion and condom use, it is important to identify regions with a low prevalence of condom use. Literature documents that factors such as level of education, employment status, region, age, living with a partner, and marital status were significantly associated with condom use (11–13). Although these valuable and relevant insights exist, changes in population dynamics and health indicators in Rwanda may have taken place in recent years. To capture these changes, this study uses a most recent dataset to quantify the spatial variations in condom use among women aged 15–49 years in Rwanda. It further implements a geo-additive model that explores spatial variations in condom use during the last sexual intercourse in Rwanda, with a focus on district-level spatial effect.

## Methods

### Study area and data

**Figure 1:**
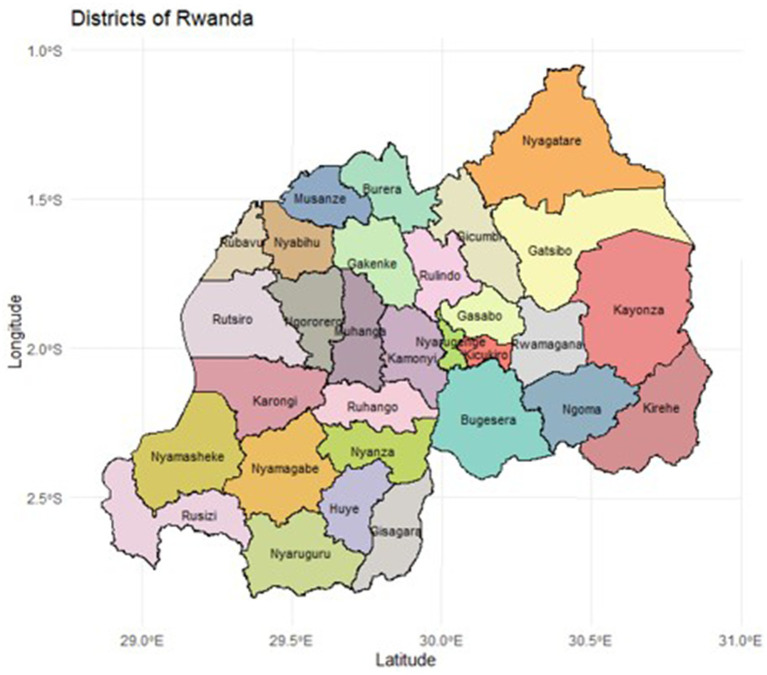
Map of Rwanda showing 30 administrative districts.

The dataset used in this study was sourced from the 2019/2020 Rwanda Demographic Health Survey (RDHS). Administratively, Rwanda is divided into four provinces (Northern, Southern, Eastern, and Western) and one city, Kigali, which serves as the capital city. These provinces are subdivided into 30 districts as shown in Figure 1. The 2012 Rwandan population census was utilized as the sampling frame for the survey, which employed a stratified, two-stage cluster sampling approach to select households for participation. The initial stage of survey design entailed choosing clusters from a list of enumeration areas (EA) that comprised the primary sampling units (PSUs). Clusters were chosen at a rate proportional to their size. The second stage of the selection procedure consisted of a systematic sampling of households from the list of households in each cluster, with the same number of households chosen from each cluster. Trained personnel went and interviewed the selected households. The DHS Sampling Manual provides an in-depth examination of sampling methodology (14). The 2019/2020 survey involved 14,634 participants from the selected households who provided informed consent. Of these, 8,481 women were eligible to participate in this study as they were aged 15–49 years and sexually active. Women who were not sexually active or who had not engaged in sexual activity within the past 12 months were excluded from the analysis. Since there were no missing values, none of the eligible participants were excluded for reasons of missing data. The 2019/2020 Rwanda DHS report contains additional information on the sampling process (15). The individual recode file from the Rwanda DHS 2019/20 was analyzed to assess factors associated with condom use during the last sexual encounter.

The outcome variable in this study was condom use during the last sexual intercourse, categorized as “Yes” or “No.” The explanatory variables included age group (15–24, 25–34, and 35–49 years), place of residence (urban or rural), region (Kigali, South, West, North, and East), and marital status, which was reclassified as living with a man (Yes or No). Other socio-demographic factors considered were highest level of education (no education, primary, secondary, and higher), employment status in the past 12 months (employed or not employed) and wealth index (poor, middle, or rich).

HIV-related variables included whether the respondent had ever tested for HIV (Yes or No), and HIV discriminatory attitudes, which were assessed using responses to four questions. Respondents who answered “No” to two stigma related questions (concern about others’ reactions if HIV positive and shame if a family member had HIV) and “Yes” to two acceptance-related questions (willingness to buy vegetables from an HIV-positive vendor and allowing HIV-positive children to attend school with HIV-negative children) were classified as having no discriminatory attitudes. Additional variables included history of STIs in the past 12 months (Yes, No, or Do not Know), and whether the respondent had experienced any form of gender-based or sexual violence, with affirmative responses to physical or sexual abuse questions classified as positive for violence exposure. Lastly, media exposure was assessed based on the frequency of reading newspapers, listening to the radio, or watching television. Respondents who accessed at least one form of media at least once a week were coded as having media exposure, while those who indicated “not at all” for all media types were classified as having no media access.

### Data availability

The data used in this study are publicly available upon request from the DHS program at: https://dhsprogram.com/.

### Ethical statement

This study was based on secondary data from the 2019/2020 Rwanda Demographic and Health Survey (RDHS). Ethical approval for the DHS data collection was obtained by the DHS Program through the Institutional Review Board (IRB) of ICF International, in compliance with the U. S. Department of Health and Human Services regulations for the protection of human subjects (45 CFR 46). Additionally, the survey protocols were reviewed and approved by the national ethics committee in Rwanda, ensuring that data collection complied with local laws, cultural norms, and ethical standards. Data collection procedures were designed to maintain confidentiality and anonymity. No personally identifiable information is included in the dataset, and all responses are kept strictly confidential.

### Statistical model

Geo-additive models transform geographically referenced responses into maps by accounting for confounding effects of various covariates. These include demographic, socioeconomic, sexual risk behaviors, and biological factors which are recognized as correlated with increased condom use cases, regardless of other environmental exposures (22). This formulation, within the frameworks of the generalized linear model (GLM) and generalized additive model (GAM), constitutes a structured additive regression model.

Denote $Y_{\mathrm{ij}}$ as the indicator for condom use by woman i in district j . If the woman used a condom during her last sexual intercourse, $P(Y_{\mathrm{ij}}=1)=p_{\mathrm{ij}}$ , and if she did not, $P(Y_{\mathrm{ij}}=0)=1-p_{\mathrm{ij}}$ . The vector $X_{\mathrm{ij}}=(x_{\mathrm{ij}1},x_{\mathrm{ij}2},\ldots .,x_{\mathrm{ijp}})\prime$ comprises of the p continuous random variables, while $Z_{\mathrm{ij}}=(z_{\mathrm{ij}1},z_{\mathrm{ij}2},\ldots ,z_{\mathrm{ijq}})\prime$ encompasses the q− categorical variables for each woman. In this study, we assume the dependent variable, $Y_{\mathrm{ij}}$ , follows a Bernoulli distribution and takes the form $Y_{\mathrm{ij}}$ | $p_{\mathrm{ij}}$ ~Bernoulli ( $p_{\mathrm{ij}}$ ), where E( $Y_{\mathrm{ij}}$ ) $=p_{\mathrm{ij}}\;$ is an unknown value linked to the covariates through the logit link function expressed in Equation 1 as follows:


(1) $$\;\text{logi}t(p_{\mathrm{ij}})=X_{\mathrm{ij}}^{\prime }\beta +Z_{\mathrm{ij}}^{\prime }\theta$$

Where *β* represents the p×1 vector of coefficients for continuous random variables, while θ stands for the q×1 - vector of coefficients for categorical random variables. To explore both the nonlinear effects of continuous random variables and spatial autocorrelation within our data, we employed a semi-parametric model utilizing a penalized regression (16). The resulting geo-additive model is expressed as:


(2) $$\;\text{logi}t(p_{\mathrm{ij}})=f_{1}(x_{\mathrm{ij}1})+\cdots +f_{p}(x_{\mathrm{ijp}})+f_{\text{spat}}(s_{j})+Z_{\mathrm{ij}}^{\prime }\theta \;$$

Where the right side in Equation 2 represents the geo-additive predictors, denoted as $f_{p}(\cdot ),$ for the nonlinear effect of continuous covariates. Additionally, $f_{\left(\text{Spat}\right)}\;$ represents a factor accounting for the spatial effect related to the location $S_{j}$ where woman ij resides. This study adopts a convolution approach toward these spatial effects, assuming that the spatial effect can be split into two distinct components: geographically organized and spatially unstructured, i.e., $f_{\text{spat}}(s_{j})=f_{\mathrm{str}}(s_{j})+f_{\text{unstr}}(s_{j}).$

The concept that proximate regions are more likely to exhibit correlated outcomes is captured by the structured spatial impact, denoted as $f_{\mathrm{str}}(s_{j})$ . According to Ngwira and Kazembe (17), the unstructured spatial impact, $f_{\text{unstr}}(s_{j})$ , addresses geographical variation attributed to the influence of unmeasured regional-level factors that lack spatial relationships.

This analysis employed a Bayesian approach for parameter estimation and inference. This framework treats all model parameters and non-linear functions as random variables, each assigned specific prior distributions. For the fixed effects, denoted as β , an independent diffuse prior P(θ)∝constant is chosen, making it a suitable choice for these parameters.

Numerous techniques are available for estimating the smooth functions, $f_{p}(\cdot )$ . A polynomial spline of degree l , applied on equal intervals t with evenly spaced knots $x_{q}^{\min}=\psi_{q_{1}}<\psi_{q2}<\ldots <\psi_{9t-1}<\psi_{q}^{\max}$ within the covariate $x_{j}$ ’s domain, is recognized for its capability to estimate the unknown smooth function f of the continuous covariate (18). In this analysis, the Bayesian penalized spline approach for the unidentified smooth function $f_{p}(\cdot )$ was adopted, utilizing P-splines, second-order random walk smoothness prior, and a third-degree spline. The proposed spline is constructed by linearly combining the basis function of g=t+l, B − spline basis function, as outlined below:


$$f_{p}(x_{p})=\sum_{p=1}^{g}\theta_{\mathrm{tp}}B_{p}(x_{p})$$

where $B_{p}(x_{p})$ represents B-splines, and $\theta_{t}$ are subjected to either a first or second-order random walk prior. The second-order random walk is characterized by the formula $\theta_{\mathrm{tp}}=2\theta_{t,p-1}-\theta_{t,p-2}+u_{\mathrm{tp}}$ , where the Gaussian errors $u_{t}$ follow a zero mean with $u_{t}\sim N(0,\delta_{t}^{2})$ . The variance parameter $\delta_{t}^{2}$ governs the smoothness of $f_{p}$ , serving as the inverse of the smoothing parameter in a frequentist approach.

In determining the spatially structured effects within the prior distribution denoted as $f_{\mathrm{str}}(s_{j})$ , this analysis employs the nearest neighbor Gaussian Markov field technique (16, 19). Regions, $s_{j}$ and $s_{k},$ sharing a common boundary are regarded as neighbours. The spatially autoregressive conditional distribution, which follows a normal distribution, is expressed as:


$$f_{\mathrm{str}}(s_{j})|f_{\mathrm{str}}(s_{k}),j\neq k,\tau_{\mathrm{str}}^{2}\sim N(\frac{\sum_{s_{k}\in \delta_{s_{j}}}f_{\mathrm{str}}(s_{k})}{N_{s_{j}}},\frac{\tau_{\mathrm{str}}^{2}}{N_{s_{j}}})$$

In this context, $N_{s_{j}}$ and $s_{k}\in \delta_{s_{j}}$ represent, respectively, the number of neighbours for the region $s_{j}$ and that region $s_{k}$ is a neighbour to region $s_{j}$ . If two regions do not share a border, they are not deemed part of the same neighborhoods. Shared borders between regions define neighborhood’s; the absence of such borders signifies non-neighboring regions. Consequently, the unweighted average of function evaluations from nearby regions $s_{k}$ constitutes to the conditional mean of $f_{\mathrm{str}}(s_{j})$ . Similarly, $\tau_{\mathrm{str}}^{2}$ regulates spatial smoothness, accommodating regional variation with proper hyperpriors to manage over-dispersion. This leads to the creation of the intrinsic conditional autoregressive (ICAR) prior distribution (16, 19). The unstructured spatial effect, $f_{\text{unstr}}(s_{j})$ , is presumed to follow a Gaussian prior distribution, modeled as $f_{\text{unstr}}\sim Ν(0,\tau_{\text{unstr}}^{2}\;)$ . Additionally, the variance hyperparameters, $\tau_{\mathrm{str}}^{2}$ and $\tau_{\text{unstr}}^{2}$ , assume an inverse gamma prior distribution, i.e., $\tau_{\mathrm{str}}^{2}\sim \mathrm{IG}(a_{j},b_{j})$ . The parameters 𝑎 and 𝑏 of the inverse gamma distribution for variance are set to 0.001, i.e., $\tau_{\mathrm{str}}^{2}\sim \mathrm{IG}(0.001,0.001)$ . This option implies an informative but weakly constraining prior, allowing the data to impact model variance. Smaller values of 𝑎 and 𝑏 result in less informative priors, providing more flexibility in variance estimates.

For comparison purposes, four models were considered:

*Model* 0: Non-spatial model.

*Model* 1: Spatially structured effect.

*Model* 2: Spatially unstructured effect.

*Model* 3: Convolutional model (with both structured and unstructured effects).

### Statistical software

The posterior distributions of model parameters were estimated using the BayesX package in R (20). Model selection was based on the Deviance Information Criterion (DIC), with the model yielding the lowest DIC considered the best fit (21, 22). To evaluate sensitivity to hyper-parameter values, we fit the model with alternative values following the approach of Gayawan et al. (23), observing minimal sensitivity to these choices. Maps illustrating the posterior mean estimates of spatial effects across Rwandan districts were created using sf packages in R version 4.3.2 and district-level shapefiles. These geospatial vector data files enabled the visual representation of condom use prevalence by district. For the structured spatial effects, an adjacency matrix ,E, was employed, representing the neighborhood structure of the 30 Rwandan districts. In this matrix, diagonal elements were set to zero, while off-diagonal elements $E_{\mathrm{jk}}\;$ indicated a shared boundary between districts *j* and *k*.

## Results

### Sample characteristics

Table 1 provides the descriptive statistics of condom use with the Chi-square test to determine significant differences in condom use at the 0.05 level. The prevalence of condom use was weighted to reflect the survey sampling weights. Out of the 8,481 eligible women, the majority were residing in rural areas (81.0%), and most participants were from the Eastern region of Rwanda (27.4%). Almost 90 % (89.9%) of the women were living with a man, and 18.9% completed a secondary education. Most participants were currently employed (83.6%), and 38.5% were from poor households. About 95.4% reported having tested for HIV before. Most women did not have HIV discriminatory attitudes (50.8%). Less than 5% reported having had an STI in the previous year (4.9%) and reported experiencing sexual violence (2.3%). In terms of access to media, only 3.3% had access to at least one of newspaper, TV or radio.

**Table 1:** Features of sexually active women aged 15–49 years who were interviewed in the 2019/20 Rwanda DHS (*n* = 8,481).

Covariate	Category	Condom use *n* (%)		Total	*p*-value
		No	Yes	*n*	
Age group	15–24	1,093 (13.2)	297 (3.3)	1,390	<0.001
	25–34	2,964 (35.1)	312 (3.9)	3,276	
	35–49	3,510 (41.0)	305 (3.6)	3,815	
Residence	Urban	1,612 (15.5)	330 (3.5)	1942	<0.001
	Rural	5,955 (73.7)	584 (7.3)	6,539	
Region	Kigali	864 (12.0)	212 (2.8)	1,076	<0.001
	South	1851 (19.1)	186 (1.9)	2037	
	West	1707 (19.1)	195 (2.2)	1902	
	North	1,251 (14.2)	113 (1.3)	1,364	
	East	1894 (24.8)	208 (2.6)	2,102	
Living with a man	Yes	7,102 (83.9)	491 (6.0)	7,593	<0.001
	No	456 (5.3)	423 (4.8)	888	
Education	No education	946 (11.3)	57 (0.7)	1,003	<0.001
	Primary	4,923 (58.0)	540 (6.3)	5,463	
	Secondary	1,316 (15.8)	258 (3.1)	1,574	
	Higher	382 (4.1)	59 (0.7)	441	
Employment	No	1,228 (14.3)	182 (2.1)	1,410	0.005
	Yes	6,339 (74.9)	732 (8.7)	7,071	
Wealth index	Poor	3,057 (35.3)	277 (3.2)	3,334	0.000
	Middle	1,500 (18.1)	121 (1.5)	1,621	
	Rich	3,010 (35.8)	516 (6.1)	3,526	
Ever tested for HIV	No	343 (3.9)	68 (0.7)	411	<0.001
	Yes	7,224 (85.4)	846 (10.0)	8,070	
HIV discriminatory attitudes	No	3,858 (45.8)	427 (5.0)	4,285	0.025
	Yes	3,709 (43.5)	487 (5.7)	4,196	
Had STI in last 12 months	No	7,212 (85.1)	845 (9.9)	8,057	0.001
	Yes	351 (4.0)	68 (0.8)	419	
	Do not know	4 (0.1)	1 (0.0)	5	
Pregnancy	No	547 (6.6)	229 (2.5)	776	<0.001
	Yes	7,020 (82.7)	685 (8.2)	7,705	
Experienced any sexual violence	No	7,390 (87.1)	899 (10.6)	8,289	0.210
	Yes	77 (2.1)	15 (0.2)	192	
Access to media	No	7,313 (86.5)	861 (10.2)	8,174	0.003
	Yes	254 (2.7)	53 (0.6)	307	

*The percentages presented are weighted. %: percent; HIV—Human Immunodeficiency Virus; GBV—gender-based violence; A Chi-squared test was conducted to determine significant difference between covariates at the 0.05 level.

In terms of condom use prevalence among the sample, the highest regional prevalence was observed in the Eastern region, where 2.6% of women reported using a condom during their last sexual intercourse. A relatively higher proportion of condom use was also reported among women residing in rural areas (7.3%). Overall, 10.8% of sexually active women aged 15–49 reported using a condom during their last sexual encounter.

### Results from a geo-additive model

Table 2 presents the adjusted posterior odds ratio (AOR) estimates and their 95% credible intervals (CrI) from the geo-additive model.

**Table 2:** Adjusted posterior odds ratio (AOR) estimates with corresponding 95% credible intervals (CrI).

Variable	Category	Non-spatial model	Unstructured spatial model	Structured spatial model	Convolutional model
		AOR [95% CrI]	AOR [95% CrI]	AOR [95% CrI]	AOR [95% CrI]
Residence	Urban	Reference	Reference	Reference	Reference
	Rural	0.88 [0.71–1.09]	0.88 [0.72–1.09]	0.89 [0.72–1.09]	0.88 [0.71–1.09]
Region	Kigali	Reference	Reference	Reference	Reference
	South	0.63 [0.48–0.86]	0.62 [0.46–0.86]	0.65 [0.47–0.95]	0.62 [0.43–0.90]
	West	0.79 [0.63–1.02]	0.80 [0.58–1.08]	0.84 [0.59–1.24]	0.78 [0.53–1.14]
	North	0.59 [0.63–0.82]	0.59 [0.44–0.84]	0.60 [0.41–0.84]	0.58 [0.40–0.86]
	East	0.70 [0.54–0.89]	0.70 [0.53–0.98]	0.71 [0.05–0.09]	0.69 [0.48–1.00]
Living with a man	No	Reference	Reference	Reference	Reference
	Yes	0.07 [0.06–0.09]	0.07 [0.06–0.09]	0.07 [0.06–0.09]	0.07 [0.06–0.09]
Education	No education	Reference	Reference	Reference	Reference
	Primary	1.38 [1.02–1.89]	1.39 [1.00–1.98]	1.39 [1.03–1.90]	1.39 [1.04–1.84]
	Secondary	1.51 [1.03–2.11]	1.52 [1.01–2.25]	1.51 [1.05–2.13]	1.51 [1.09–2.06]
	Tertiary	1.24 [0.82–1.90]	1.25 [0.97–1.91]	1.24 [0.83–1.91]	1.28 [0.78–2.02]
Employment	No	Reference	Reference	Reference	Reference
	Yes	1.14 [0.94–1.37]	1.13 [0.91–1.39]	1.13 [0.91–1.38]	1.14 [0.94–1.41]
Wealth index	Poor	Reference	Reference	Reference	Reference
	Middle	0.85 [0.67–1.09]	0.85 [0.66–1.07]	0.85 [0.64–1.10]	0.83 [0.67–1.09]
	Rich	1.47 [1.21–1.77]	0.85 [0.65–1.07]	1.48 [1.17–1.83]	1.45 [1.17–1.77]
Ever tested for HIV	No	Reference	Reference	Reference	Reference
	Yes	1.19 [0.83–1.39]	1.19 [0.84–1.75]	1.17 [0.87–1.71]	1.12 [0.85–1.52]
HIV discriminatory attitudes	No	Reference	Reference	Reference	Reference
	Yes	0.99 [0.84–1.16]	0.99 [0.87–1.16]	0.99 [0.84–1.15]	0.99 [0.84–1.16]
Had an STI in the last 12 months	No	Reference	Reference	Reference	Reference
	Yes	1.37 [0.96–1.83]	1.41 [1.02–1.90]	1.40 [1.02–1.95]	1.39 [1.03–1.87]
	Do not know	0.45 [0.03–5.10]	0.30 [0.00–4.90]	0.43 [0.01–5.77]	0.40 [0.01–5.46]
Pregnancy	No	Reference	Reference	Reference	Reference
	Yes	0.92 [0.70–1.17]	0.89 [0.70–1.14]	0.90 [0.69–1.15]	0.89 [0.71–1.15]
Ever experienced any sexual violence	No	Reference	Reference	Reference	Reference
	Yes	1.25 [0.72–2.10]	1.26 [0.68–2.07]	1.24 [0.69–2.06]	1.24 [0.66–2.18]
Access to media	No	Reference	Reference	Reference	Reference
	Yes	1.03 [0.68–1.50]	1.04 [0.70–1.48]	1.02 [0.69–1.55]	1.03 [0.71–1.56]

### Results of the geo-additive model for condom use

#### Model selection

Table 3 presents the results of the DIC and the effective number of parameters, pD , for each of the fitted models. In this analysis, Model 3, which includes both structured and unstructured spatial effects, linear fixed effects, and the non-linear effect of continuous age, yielded the lowest DIC value (4795.37), indicating the best fit for the data, even though it is the most complex with the highest pD(32.11) . Consequently, the discussion of results of this study are based on Model 3, which provides a comprehensive understanding of the factors influencing condom use in Rwanda by integrating spatial effects and the non-linear influence of age.

**Table 3:** Model fit comparisons.

Model	Deviance information criterion	Effective number of parameter
Model 0	4798.23	21.48
Model 1	4796.87	30.73
Model 2	4799.77	28.73
Model 3	4795.37	32.11

### Fixed effects

Results from Model 3 in Table 2 show that women residing in the South region had significantly lower odds of condom use compared to those residing in Kigali (AOR = 0.62, 95% CrI: 0.43–0.90). Similarly, women in the North region had significantly lower odds of condom use than those in Kigali (AOR = 0.58, 95% CrI: 0.40–0.86). Women who reported living with a man had significantly lower odds of condom use compared to those not living with a man (AOR = 0.07, 95% CrI: 0.06–0.09). This suggests a strong association between not living with a man and an increased likelihood of condom use. Women with primary education had significantly higher odds of condom use compared to those with no education (AOR = 1.39, 95% CrI: 1.04–1.84). This suggests that having some level of formal education is positively associated with condom use. Similarly, women with secondary education had significantly higher odds of using condoms compared to those with no education (AOR = 1.51, 95% CrI: 1.09–2.06), suggesting a positive association between secondary education and condom use. Women classified as wealthy (rich wealth index) had significantly higher odds of using condoms compared to those classified as poor (AOR = 1.45, 95% CrI: 1.17–1.77), indicating that wealthier women are more likely to use condoms than poorer women.

Women who reported having had an STI in the last 12 months had significantly higher odds of condom use compared to those who did not report an STI (AOR = 1.39; 95% CrI: 1.03–1.87). This suggests that having experienced an STI in the past 12 months is associated with an increased likelihood of using condoms, possibly as a preventive measure against further infections.

### Non-linear and spatial effect

Table 4 presents the posterior mean and 95% credible interval (CrI) for the variance components of the smooth terms, including the non-linear effect of age and spatial effects in the model. In this context, the precision of an effect is the inverse of its variance, meaning higher precision corresponds to lower variance, while greater variance indicates more variability in the effect across individuals or geographic areas. The non-linear effect of a woman’s age in years $(\tau_{r}^{2})\;$ had a mean–variance of 0.0049, with a 95% CrI of [0.0006, 0.0231]. This suggests that although a non-linear effect exists, its variability across women of different ages is relatively small.

**Table 4:** Posterior mean and 95% credible intervals (CrI) for variance components and spatial effect precisions (non-linear, structured, and unstructured).

Variable	Mean	95% CrI
*Non-linear effect*		
Woman’s age in years $\left(\tau_{r}^{2}\right)$	0.0049	[0.0006–0.0231]
*Spatial effect*		
Structured spatial effect $\left(\tau_{\mathrm{str}}^{2}\right)$	0.0274	[0.0006–0.1327]
Unstructured spatial effect $\left(\tau_{\text{unstr}}^{2}\right)$	0.0174	[0.0009–0.0551]

For the spatial effects, the model accounted for both structured and unstructured spatial variability. The precision of the structured spatial effect $(\tau_{\mathrm{str}}^{2})\;$ was 0.0274, with a 95% CrI of [0.0006, 0.1327], while the precision of the unstructured spatial effect $(\tau_{\text{unstr}}^{2})\;$ was 0.0174, with a 95% CrI of [0.0009, 0.0551].

Although both spatial effects show some variability, the structured spatial effect exhibits higher precision (indicating lower variance) than the unstructured spatial effect. However, the difference in precision between the two effects is less pronounced compared to other studies, such as Kazembe et al. (24). This suggests that both the structured and unstructured spatial effects similarly contribute to explaining the variation in condom use among women in Rwanda, though the structured spatial effect appears to play a slightly more dominant role.

**Figure 2:**
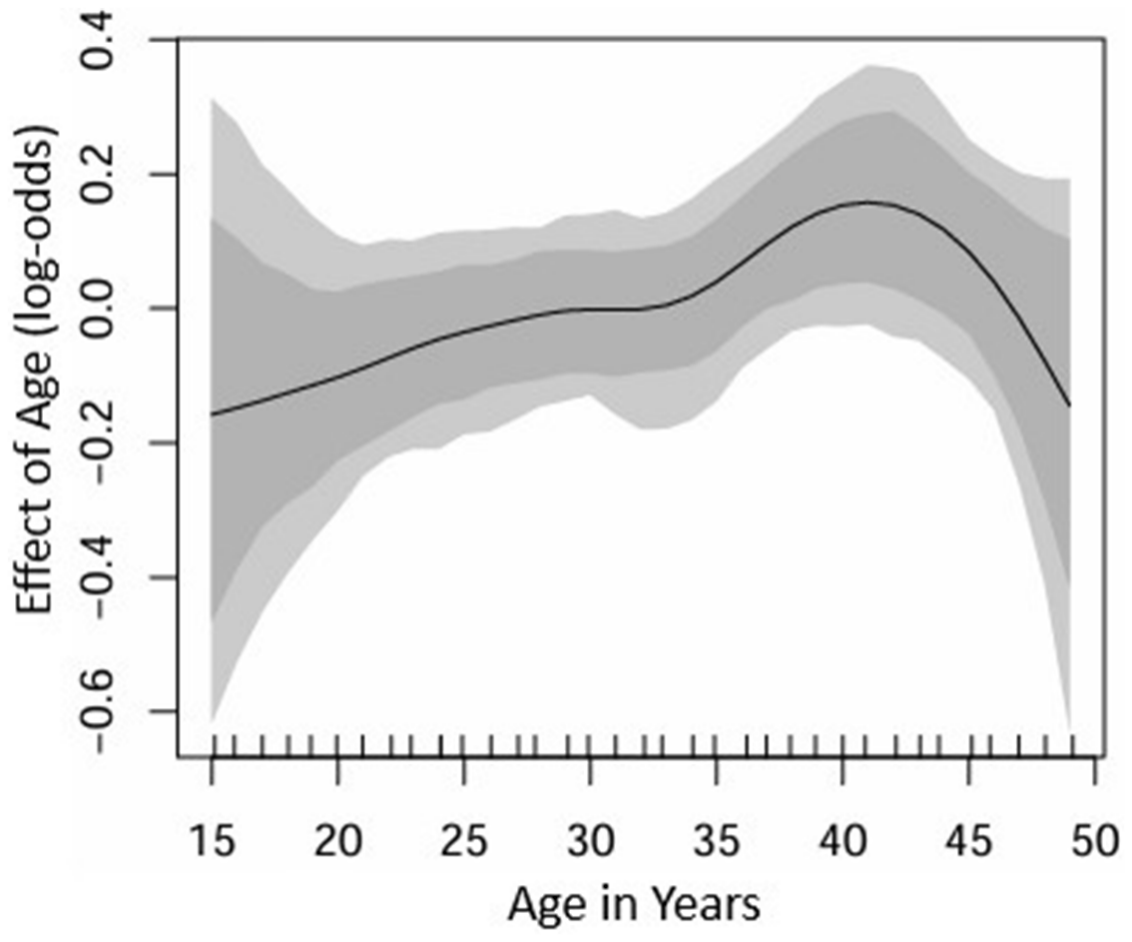
Estimated non-linear effects of a woman’s age in years on the log-odds of condom use. The posterior mean and corresponding 95% credible intervals are displayed.

Figure 2 illustrates the non-linear effect of a woman’s age in years on the log-odds of condom use, along with the 95% credible intervals. The solid line represents the posterior mean effect, while the shaded areas indicate the 95% credible intervals. The effect shows an initial increase in condom use from around age 15 to approximately 30, suggesting a higher likelihood of condom use among younger and middle-aged women. After age 30, the effect stabilizes and remains consistent until around age 35, followed by another increase up to age 40. Beyond this point, the likelihood of condom use begins to decline, particularly among women over 40, indicating a lower probability of condom use in older age groups. If a linear effect were assumed, it would have overestimated the impact of age, especially for women in their 30s and 40s, failing to capture the subtle variation in condom use behavior across different age groups. This pattern aligns with the method that Roberts et al. (25) used, emphasizing the importance of modeling age as a non-linear effect to accurately capture its varying influence on condom use across different life stages (25).

**Figure 3:**
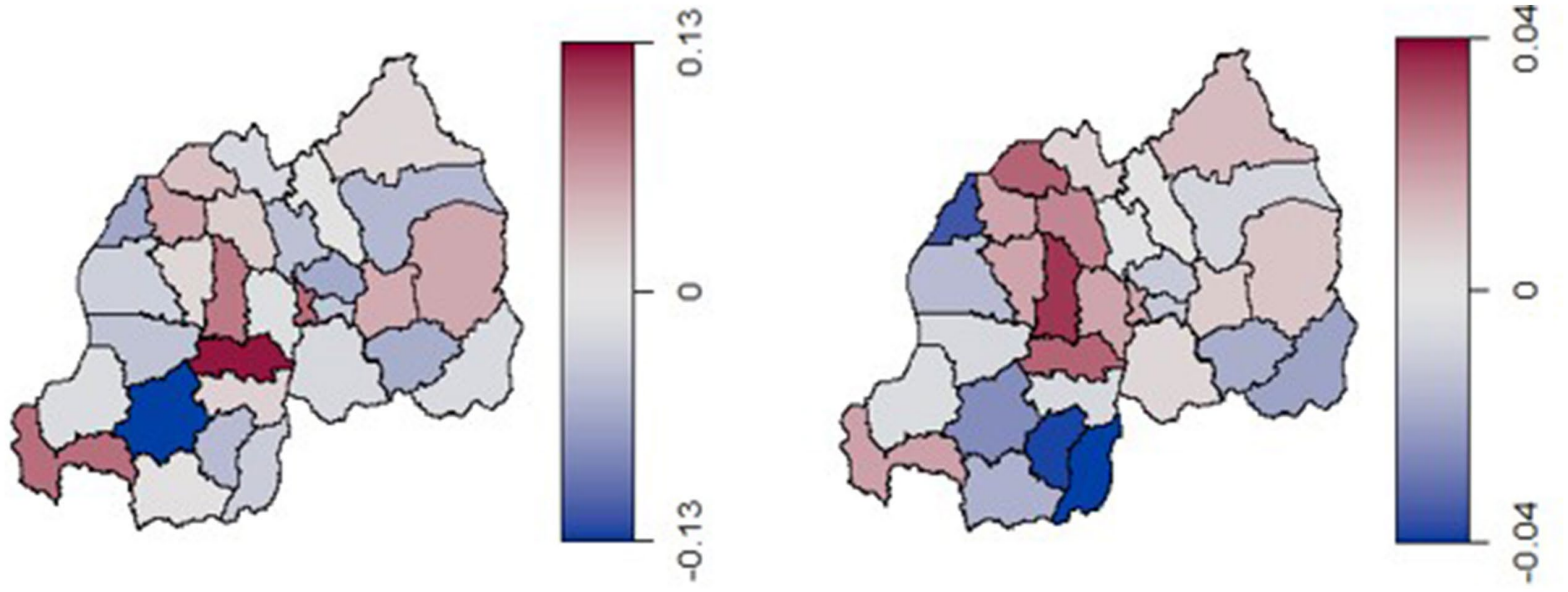
Estimated posterior means of the unstructured spatial effect (left) and the structured spatial effect (right) on the log-odds of condom use.

Figure 3 depicts the estimated posterior means for unstructured (left) and structured (right) spatial effects on condom use across Rwandan districts. In these maps, blue districts represent areas with a negative spatial effect (lower odds of condom use), while maroon districts indicate a positive spatial effect (higher odds of condom use). The structured spatial effects reflect the influence of spatially correlated factors, while the unstructured spatial effects capture district-specific local variations.

The structured spatial effects ranged between −0.04 and 0.04, indicating only modest variability and suggesting weak spatial correlations in condom use across Rwanda. Districts in the central-to-northern regions, such as Ruhango, Muhanga, and Musanze, exhibit stronger structured spatial effects, indicating the presence of regionally correlated factors influencing condom use behavior in these areas. In contrast, districts in the western and southern regions, such as Nyamagabe, Huye, and Gisagara, show lower structured spatial effects, suggesting a weaker influence of these broader spatial factors.

The unstructured spatial effects, with values ranging from −0.13 to 0.13, reflect independent district-level variations. These unstructured effects suggest that local factors, specific to individual districts, play a more significant role in determining condom use in regions like Ruhango and Rusizi which show higher positive unstructured effects. Conversely, districts such as Nyamagabe and Gasabo, display negative unstructured effects, indicating lower odds of condom use that are not linked to broader spatial patterns.

**Figure 4:**
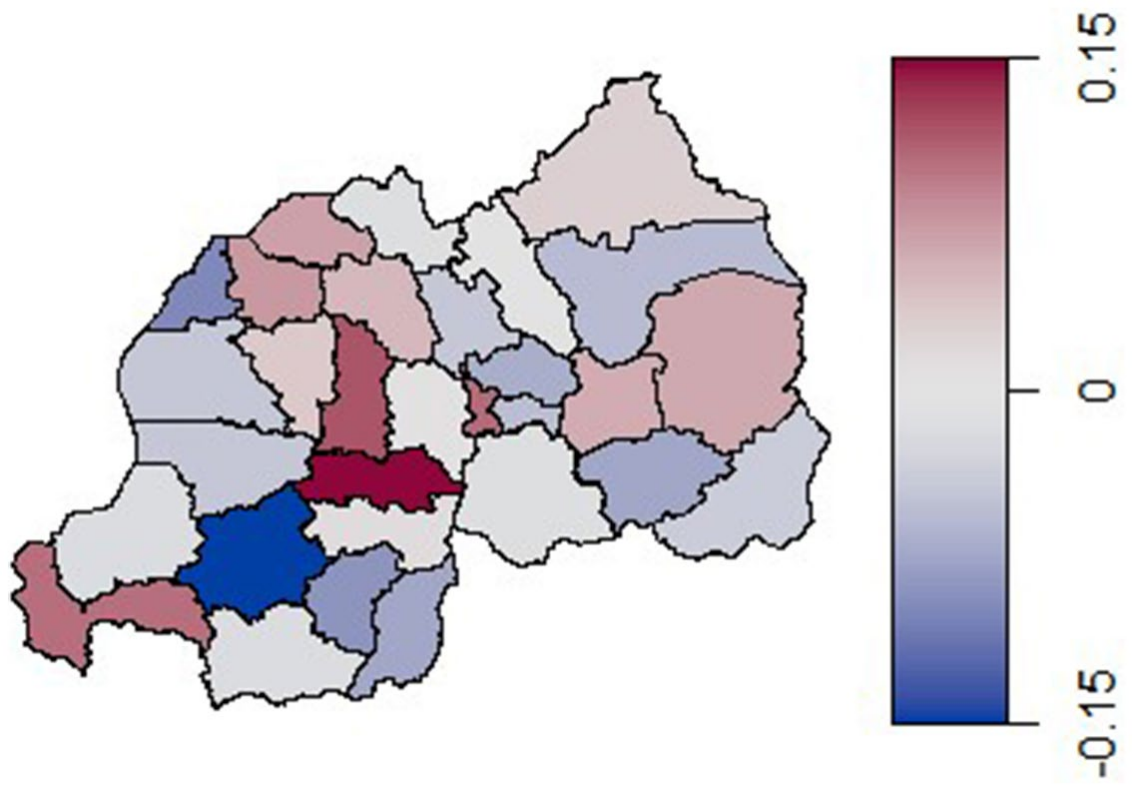
Estimated posterior means of the total spatial effect (both the unstructured and the structured spatial effects) on the log-odds of condom use.

Additionally, certain districts in the central region, such as Muhanga, Ruhango, Kamonyi, and Nyarugenge show a stronger association with structured spatial effects. This may reflect the influence of regionally correlated factors such as health services, cultural practices, and socio-economic conditions that impact sexual health behaviors. Figure 4 illustrates the estimated posterior means for the total spatial effect (a combination of both unstructured and structured spatial effects) on the log-odds of condom use across districts in Rwanda. The map reveals regional patterns in condom use among reproductive-aged women in Rwanda. Districts in the western and central regions, particularly those near Lake Kivu like Rubavu and Rutsiro, exhibit strong negative spatial effects (shaded in blue), indicating lower condom use prevalence.

In contrast, a central district shaded in dark red shows a highly positive spatial effect, suggesting a strong association with higher condom use rates. The eastern and northern regions generally display lighter or neutral colors, implying minimal or no spatial effect on condom use prevalence. These findings highlight a localized variation, with the central regions showing a tendency toward higher condom use rates.

## Discussion

We applied a hierarchal Bayesian spatial model to examine the risk factors and spatial variation in condom use among Rwandan women aged 15–49. This model enables us to assess and visualize the residual spatial effects on condom use while accounting for the influence of other covariates. Furthermore, it enables the investigation of nonlinear relationships in continuous variables. In our research, incorporating the spatial effect resulted in a lower DIC, indicating better model fit.

The findings from this analysis on condom use among Rwandan women align with previous research, showing that condom use varies significantly based on socio-demographic and spatial factors. In line with other research on spatial influences on reproductive health behaviors (24, 26), the structured spatial effects observed here suggest that regionally correlated factors significantly influence condom use behaviors. This aligns with findings by Adebayo et al. (26), who modeled geographical variations in modern family planning use in Nigeria and observed similar patterns of regionally associated effects (26), indicating that localized cultural, economic, and accessibility factors might also play a role in shaping family planning and condom use behavior across different districts in Rwanda. For example, in regions near Lake Kivu, certain district-specific factors such as access to health services and cultural practices appear to impact condom use, as shown in studies exploring spatial health outcomes (24).

This resemblance with findings from (26), who also applied Bayesian spatial modeling to analyse HIV-related behaviors across Nigerian regions, underscores that spatial factors in both structured and unstructured forms can reveal localized influences on condom use, despite modest effects in some areas. In Rwanda, the structured spatial effects, while less pronounced, still suggest localized cultural norms or socio-economic conditions influencing sexual health behaviors in certain districts.

The observed non-linear effect of age, with condom use peaking around age 40, mirrors similar findings in studies that have highlighted age-specific health behavior patterns (25). The non-linear modeling approach reveals subtle variations in condom use by age, which a linear model might overlook. For instance, Roberts et al. (25) showed that non-linear modeling is crucial to capture the nuances in age-related health behaviors, underscoring the importance of this approach for accurate analysis.

Comparing structured and unstructured spatial effects reveals that unstructured effects contribute as significantly as structured effects in explaining district-specific condom use variations. For instance, districts like Ruhango show high positive unstructured effects, indicating district-level factors that may influence condom use behaviors. This pattern aligns with findings from studies in other Sub-Saharan African contexts (17), identifying substantial local variations in health behaviors. However, unlike studies where structured effects were more dominant, the relatively balanced influence between structured and unstructured effects in Rwanda suggests that broader region-wide influences on condom use may be less pronounced (24).

### Strengths and limitations

This study presents several strengths that contribute to its robustness and relevance. The use of the most recent and nationally representative RDHS 2019/20 ensures that the findings reflect current condom use behaviors among reproductive aged women in Rwanda. Secondly, the application of a geo-additive Bayesian spatial model allows for the exploration of both structured and unstructured spatial effects while controlling for relevant covariates, providing detailed insight into regional variations in condom use. This spatial analysis at the district level offers a valuable contribution to the literature by identifying high and low prevalence areas, which can guide region specific interventions.

However, some limitations must be acknowledged. As this study is based on cross-sectional data, it does not allow this study to determine the trend of condom use prevalence over the years. There is also potential for recall bias, as participants may have misremembered past sexual behavior or misunderstood sensitive questions.

### Implications for policy and practice

The findings of this study have several important implications for policy and practice. Firstly, the identified spatial disparities in condom use underscore the need for region specific public health interventions. Policymakers and program implementers should prioritize districts such as Nyamagabe and Gasabo, which showed low condom use, by tailoring sexual health education, service delivery, and outreach programs to local contexts and barriers.

Therefore, the findings highlight the need for sustained investment in behavior change communication, partner negotiation skills training, and improved condom accessibility, particularly among women in cohabiting relationships, where condom use was found to be exceptionally low. Addressing cultural norms, power dynamics, and trust issues within relationships could significantly improve consistent condom use.

## Conclusion

This balance between structured and unstructured effects implies that interventions targeting condom use in Rwanda might be more effective if tailored to specific districts rather than implemented on a larger regional scale, an insight supported by previous spatial health studies that emphasize localized intervention strategies in regions with similar socio-cultural heterogeneity.

## Data Availability

The datasets presented in this study can be found in online repositories. The names of the repository/repositories and accession number(s) can be found in the article/supplementary material.
